# Outcome of pregnancy in women with splenomegaly

**DOI:** 10.1186/s12884-023-05465-0

**Published:** 2023-03-04

**Authors:** AbdelAziem A. Ali, Israa Badr Eldin

**Affiliations:** 1 Department of Obstetrics and Gynecology, Faculty of Medicine, Kassala University, Kassala, Sudan; 2 Alshuhada Teaching Hospital, Khartoum, Sudan

**Keywords:** Splenomegaly, Pregnancy, Obstetric, Lymphocyte, Sudan

## Abstract

**Background:**

The spleen is a lymphopoietic organ, contains almost one quarter of the body’s lymphocytes.

**Method:**

This was a prospective cross sectional study, carried out at Kassala hospital, Sudan between 1st of May 2019 to 30th of April 2020. The objective of this study was to investigate the outcome of pregnancy in women with splenomegaly. A total coverage of 57 women with splenomegaly were approached among all pregnant women attending the hospital and asking for care. An enlarged spleen detected by palpation and subcategorized into mild, moderate and severe one according to its length below the left costal margin using Ultrasound. Data was collected using structured questionnaire. Means and proportions were compared between the groups of the study-using student and *x*^2^ test, and *P* < 0.05 was considered significant.

**Results:**

The most predominant type of splenomegaly was massive (50.9%) splenomegaly. The reported obstetric complications among the investigated women include: intrauterine growth restriction (19.3%), preterm labor ((17.5%), miscarriage (12.3%) and stillbirth (3.5%). Out of 50 patients their pregnancy progressed to delivery, three patients developed primary hemorrhage requiring blood transfusion with ≥ 2 units of blood. Respiratory distress syndrome (RDS), acute tachypnea of the newborn and stillborn babies were observed in 18%, 6% and 4% respectively. Higher proportion of women with poor obstetric outcomes was reported in cases of massive splenomegaly in comparison with other types.

**Conclusion:**

The study showed significant association between adverse obstetric outcomes and massive splenomegaly. Thus, it is important to consider splenomegaly as one of the factors making the pregnancy high-risk one.

## Background

The spleen is the largest lymphoid organ measures 1 × 3 × 5 inches and lies between the 9th and 11th ribs [1]. Splenomegaly is defined as spleen enlargement and it differs from hypersplenism, which refers to hyperactive spleen [2]. Splenomegaly is non-specific clinical findings and it is usually found in infectious diseases (malaria, leishmania, shistosomiasis), hematological disorders (sickle cell disease, hemoglobinopathy), spleen infiltration with cancer, portal hypertension, immunological problem, hepatic cirohosis, metabolic diseases (amyloidosis and Gaucher disease) and extramedullary hematopoiesis [2, 3]. The spleen contains almost one quarter of the body’s lymphocytes, helps mediate both cellular and humoral immunity, and participates in immune responses against blood-borne pathogens [4]. It also acts as a filter and removes the damaged red blood cells (RBCs) and opsonized bacteria from the circulation [5]. Despite of little data, which is available in the literature concerning the obstetric outcomes in patients with splenomegaly, there is an evidence of significant association of massive splenomegaly, anemia, fever and chronic abdominal pain during pregnancy [1]. A gain splenomegaly might be a predictor for poor obstetric outcome in particular intrauterine growth restriction [1].

In Kassala, east Sudan, there is a high frequency of maternal morbidities and mortality which needs improvement in obstetric care. In Kassala, the obstetric complications included: pre-term birth (2.6%); pre-eclampsia/eclampsia (4.2%) and haemorrhage (2.9%) and caesarean delivery rate is 31.1%. While 89.4% of the newborn babies were taken home, 6% were admitted to the nursery, 4.4% were stillbirths, and 0.2% experienced immediate neonatal deaths [6]. Thus, we planned to investigate the outcome of pregnancy among women with splenomegaly in eastern Sudan aiming to provide the caregivers and health planners with basic data necessary for intervention.

## Methods

This was a cross sectional facility based study carried out in the period between 1st of May 2019 to 30th of April 2020. A total coverage of 57 pregnant women with enlarged spleen at Kassala hospital, eastern Sudan were approached to act as source for data in this study. This sample was determined among all pregnant women who attended the hospital and asking for antenatal care with no exclusion criteria a part from patient refusal. Aiming to investigate the outcomeof pregnancy in women with splenomegaly, an enlarged spleen detected by palpation during the physical examination and subcategorized into mild, moderate and severe one. Ultrasound was performed to help determine the size of the spleen. Mild splenomegaly is when the spleen is palpable < 5 cm below the left costal margin and maximum cephalocaudal splenic diameter is < 10 cm [7]. Moderate splenomegaly is when the spleen is palpable 5–8 cm below left costal margin with the maximum cephalocaudal splenic diameter being 11-20 cm [7]. The massive splenomegaly is when the spleen is palpable > 8 cm below the left costal margin with the maximum cephalocaudal diameter of spleen being > 20 cm [7]. The data was collected using structured questionnaire dealing with the different variables. Information sought in the questionnaire included socio-demographic characteristics (age, residence), obstetrical data (parity, antenatal care utilization), the presenting complaint, medical history (anemia, deep vein thrombosis, diabetes mellitus, hypertension, thrombophylia, nephritic syndrome……ect) and maternal outcome (improvement, residual impairment and death). Proper systemic examination was performed to each patient by physician including cardiovascular, respiratory, abdomen, musculoskeletal and central nervous systems. Baseline investigations were performed for every patient and repeated when clinically indicated. These include blood tests, such as a complete blood count to check the number of red blood cells, white blood cells and platelets, blood urea, serum creatinine, and serum bilirubin. All patients were under multidisciplinary care and closely followed up to six weeks following the delivery.

### Data analysis

Data was analyzed using Statistical Package for Social Sciences (SPSS) for Window version 21 (SPSS Inc., Chicago, IL, USA) and double checked before analysis. Means and proportions for the socio-demographic characteristics were compared between the groups of the study-using student and *x*^2^ test, respectively and *P* < 0.05 was considered significant.

## Results

### General characteristics

During the study period 57 patients with splenomegaly among 41,682 pregnant women presented to Kassala hospital, yielding an incidence rate of 1.3/1000 pregnant women. The mean age (± SD) of the investigated women was 31.12 (± 4.8). The majority (52.6%) of these patients had high parity (≥ 5), illiterate (69%), of rural residence (75%) and unbooked (92.8%). Among the study group 49 patients (86%) were housewives, 7 (12.3%) non skilled workers and 1(1.7%) an employee. Thirty-five patients (61.4%) were diagnosed prior to the current pregnancy and the other 22 (38.6%) patients were diagnosed for the first time during the current pregnancy.

### Causes and types of splenomegaly

In this study, the identified causes of splenomegaly were: tropical splenomegaly (52.7%), portal hypertension (26.3%), tuberculosis (8.8%), leishmaniosis (5.3%), idiopathic thrombocytopenia (3.5%) and unidentified cause (3.5%). The most predominant type of splenomegaly was massive (50.9%) splenomegaly followed by mild (31.6%) and moderate (17.5%) ones and none of all patients needed splenectomy.

### Hematological findings

Regarding the hematological findings among the investigated women: Anemia was the most common complication and it was seen in all patients. Severe anemia was seen in 36.8% of the patients and received packed cell. Thrombocytopenia was the next common complication seen in (22.8%) of the patients, four (7%) patients had severe thrombocytopenia (platelet < 50,000cells/mm3) and received platelets transfusion. Leucopenia (WBC count < 4000 × 109 cells/ L) was reported in 3.5%. It worth mention that among those who had severe thrombocytopenia one patient presented with epistaxis.

### Outcome of pregnancy among the investigated women

With regard to the outcome of pregnancy among the patients with splenomegaly, the mean gestational age at delivery was 38 weeks and the mean birth weight was 2.7 Kg. Again the reported obstetric complications among the investigated women include: intrauterine growth restriction (19.3%), preterm labor ((17.5%), miscarriage (12.3%) and stillbirth (3.5%). Out of 50 patients their pregnancy progressed to delivery, two patients underwent cesarean delivery due to fetal distress and meconium stained liquor in early labor. Three patients developed primary hemorrhage requiring blood transfusion with ≥ 2 units of blood one of them had disseminated coagulopathy. Most of the delivered babies (36/50, 72%) had normal Apgar scoring. However, respiratory distress syndrome (RDS), acute tachypnea of the newborn and stillborn babies were observed in 18%, 6% and 4% respectively. In this study severe form of anemia and severe thrombocytopenia, were significantly varied among the different types of splenomegaly (more common among the group of massive splenomegaly). Again higher proportion of women with poor obstetric outcomes was reported in cases of massive splenomegaly in comparison with other types, Tables 1 and 2. Using ANOVA analysis there was no significant variation in maternal and perinatal outcomes when we compared the different groups of patients based on the underlying causes of splenomegaly, Table 3.

**Table 1:** Obstetric outcomes among the three groups of splenomegaly

Variables	Mild Splenomegaly (n = 18)	Moderate Splenomegaly (n = 10)	Massive Splenomegaly (n = 29)	*P*
Stillbirth	1 (5.5%)	0(100%)	1(3.4%)	0.110
Miscarriage	0(0.0)	1(10%)	6(20.6%)	0.032
Preterm birth	1 (5.5%)	1 (10%)	8(27.6%)	0.011
IUGR	0 (0.0)	1 (10%)	10(34.5%)	0.001
Hemorrhage	0 (0.0)	0 (0.0)	3(10.3%)	0.025
DIC	0 (0.0)	0 (0.0)	1 (3.4%)	0.614

**Table 2:** Perinatal outcomes among the three groups of splenomegaly

Variables	Mild Splenomegaly (n = 18)	Moderate Splenomegaly (n = 10)	Massive Splenomegaly (n = 29)	*P*
Normal Apgar Score	12 (66.6%)	9(90%)	15(51.7%)	0.005
RDS	1(5.5%)	2(20%)	6(20.7%)	0.132
ATN	0 (0.0)	1 (10%)	2(6.9%)	0.411

Abbreviation: RDS: Respiratory Distress Syndrome; ATN: Acute Tachypnea of the Newborn

**Table 3:** Obstetric outcomes among the different groups of women with splenomegaly according to the causing disease in Kassala hospital, eastern Sudan

Variables	TSS (30)	Portal HTN (15)	TB (5)	Leishmaniosis(3)	ITP(2)	Unknown(2)	*P*
IUGR	6(20)	3 (20)	1(20)	1(33)	0 (0.0)	0 (0.0)	0.6
Preterm birth	5(17)	3 (20)	1(20)	1(33)	0 (0.0)	0 (0.0)	0.2
Miscarriage	3(10)	2 (13)	1(20)	0 (0.0)	0 (0.0)	1(50)	0.05
Stillbirth	1(3)	0 (0.0)	0 (0.0)	0 (0.0)	1(50)	00	0.06
Hemorrhage	2(6)	1(6)	0 (0.0)	0 (0.0)	0 (0.0)	0 (0.0)	0.1
RDS	5(17)	2(13)	1(20)	1(33)	0 (0.0)	0 (0.0)	0.8
ATN	1(3)	1(6)	1(20)	0 (0.0)	0 (0.0)	0 (0.0)	0.07

Abbreviation: RDS: Respiratory Distress Syndrome; ATN: Acute Tachypnea of the Newborn

## Discussion

Splenomegaly is an important sign and challenging medical problem that associated with an underlying disease. In this study tropical splenomegaly (52.7%), portal hypertension (26.3%), tuberculosis (8.8%), leishmaniasis (5.3%) and idiopathic thrombocytopenia (3.5%) were the identified causes for splenomegaly. In line with our study hyper-reactive malarial splenomegaly (HMS; historically known as Tropical Splenomegaly Syndrome” [TSS] or “Big Spleen Disease”) is one of the most common causes of a massive splenomegaly in malaria-endemic areas [8, 9]. Sudan is an endemic area of malaria and other infectious diseases in which enlarged spleen is one of their manifestation [10, 11]. The incidence of splenomegaly mainly depends on the geographical location reflecting the etiology since the causes may vary with diseases prevalent in a given area [1]. In Asia and Africa, tropical splenomegaly is mainly caused by malaria, sickle cell disease and schistosomiasis [1]. Splenomegaly during pregnancy present a further testing situation to the obstetrician. The diagnosis especially during advanced pregnancy becomes difficult by clinical examination. There are few data in the literature addressing splenomegaly in pregnancy. However, the risk of splenomegaly in pregnancy in terms of anemia, thrombocytopenia and increased susceptibility to infection, and the anxiety are reported outcomes [9]. Massive splenomegaly might cause some discomfort by the growing uterus. In addition, the pregnancy may be a factor that aggravate the complications associated with hyperactive spleen. Pregnant women are more susceptible to malaria and, in Sudan, pregnant women are susceptible to Plasmodium falciparum malaria irrespective of age or parity, and malaria is a leading cause of maternal and perinatal morbidity and mortality [11]. Other infectious disease that contribute to enlarged spleen like shistosomiasis and tuberculosis are also highly prevalent in the study area [12, 13]. The complications of splenomegaly in pregnancy in terms of anemia, thrombocytopenia, increases the susceptibility to poor pregnancy outcomes. Anemia is a reported cause for stillbirth, preterm labor, preeclampsia and postpartum hemorrhage [14]. The significant association between massive splenomegaly and these outcomes logically explained by the exclusive occurrence of severe anemia in this type of enlarged spleen. *Allen* (1924) reported two cases of splenic anemia complicating pregnancy, the first patient died suddenly during delivery [15]. In this study, we reported many obstetric complications among the investigated women who suffered enlarged spleen. Therefore, splenomegaly in pregnancy requires specific and particular care to improve both maternal and perinatal outcome.

Sudan, including the study area, is characterized by high rates of maternal and perinatal mortality [6]. Maternal anemia was beyond these high rates of deaths. Therefor without addressing splenomegaly, anemia and all the tropical diseases, we cannot reach an acceptable improvement in maternal health care.

## Conclusion

Our observations indicate that splenomegaly during pregnancy in endemic area of infectious disease such as malaria and tuberculosis call for urgent intervention. The results failed to find any association between the outcome of pregnancy and the underlying causes of splenomegaly. However, there is considerable adverse outcome during pregnancy among women with splenomegaly and our results showed significant association between these outcomes and massive splenomegaly.

## Recommendation

There are limitations to this study: the sample size was small reflecting the relative rarity of splenomegaly during pregnancy also it is a facility based study and not reflect the burden of the problem in the whole community thus more research is needed in this topic. Enlargement of the spleen during pregnancy should be considered as one of the factors making the pregnancy high-risk one.

## Data Availability

Relevant data generated or analyzed during this study are available from the corresponding author on reasonable request.
